# Hybrid Membrane‐Coated Nanoparticles for Precise Targeting and Synergistic Therapy in Alzheimer's Disease

**DOI:** 10.1002/advs.202306675

**Published:** 2024-04-22

**Authors:** Rong‐Rong Lin, Lu‐Lu Jin, Yan‐Yan Xue, Zhe‐Sheng Zhang, Hui‐Feng Huang, Dian‐Fu Chen, Qian Liu, Zheng‐Wei Mao, Zhi‐Ying Wu, Qing‐Qing Tao

**Affiliations:** ^1^ Department of Neurology and Research Center of Neurology in Second Affiliated Hospital, and Liangzhu Laboratory Zhejiang University School of Medicine Hangzhou 310009 China; ^2^ MOE Key Laboratory of Macromolecular Synthesis and Functionalization Department of Polymer Science and Engineering Zhejiang University Hangzhou 310027 China; ^3^ MOE Frontier Science Center for Brain Science and Brain‐Machine Integration School of Brain Science and Brain Medicine Zhejiang University Hangzhou 310058 China; ^4^ CAS Center for Excellence in Brain Science and Intelligence Technology Shanghai 200031 China

**Keywords:** Alzheimer's disease, hybrid cell membrane‐coated liposomes, inflammatory targeting, synergistic therapy

## Abstract

The blood brain barrier (BBB) limits the application of most therapeutic drugs for neurological diseases (NDs). Hybrid cell membrane‐coated nanoparticles derived from different cell types can mimic the surface properties and functionalities of the source cells, further enhancing their targeting precision and therapeutic efficacy. Neuroinflammation has been increasingly recognized as a critical factor in the pathogenesis of various NDs, especially Alzheimer's disease (AD). In this study, a novel cell membrane coating is designed by hybridizing the membrane from platelets and chemokine (C–C motif) receptor 2 (CCR2) cells are overexpressed to cross the BBB and target neuroinflammatory lesions. Past unsuccessful endeavors in AD drug development underscore the challenge of achieving favorable outcomes when utilizing single‐mechanism drugs.Two drugs with different mechanisms of actions into liposomes are successfully loaded to realize multitargeting treatment. In a transgenic mouse model for familial AD (5xFAD), the administration of these drug‐loaded hybrid cell membrane liposomes results in a significant reduction in amyloid plaque deposition, neuroinflammation, and cognitive impairments. Collectively, the hybrid cell membrane‐coated nanomaterials offer new opportunities for precise drug delivery and disease‐specific targeting, which represent a versatile platform for targeted therapy in AD.

## Graphical abstract

A novel cell membrane coating is designed by hybridizing the membranes from platelets and chemokine receptor 2 overexpressing cells to cross the blood brain barrier (BBB) and target neuroinflammatory lesions. Rapamycin and 1‐trifluoromethoxyphenyl‐3‐(1‐propionylpiperidin‐4‐yl) urea (TPPU) are loaded into the liposomes to achieve a synergistic therapy. These drug‐loaded hybrid cell membrane‐coated liposomes precisely target specific lesions and ameliorate cognitive deficiency in Alzheimer's disease.

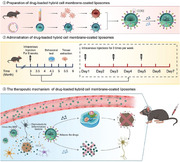

## 1 Introduction

The blood brain barrier (BBB) is a critical component of the central nervous system (CNS) that plays a crucial role in regulating the transport of various substances, including drugs, into and out of the brain.^[^ ^1^ ^]^ Although the BBB is essential for maintaining proper functioning of the CNS, it also poses challenges for the treatment of neurological disorders (NDs). The emergence of nanotechnology has largely overcome this problem. However, most strategies are based on chemical engineering modifications, which may lead to poor biocompatibility.^[^ ^2^, ^3^, ^4^, ^5^ ^]^ Few studies have designed cell membrane‐coated nanoparticles to enhance compatibility and safety.^[^ ^6^ ^]^ The potential of using platelet membranes as transporters is attractive, due to their ability to flow through destructive blood vessels, concurrently elongating circulation duration.^[^ ^7^, ^8^ ^]^

In addition to single‐cell membrane‐coated nanotechnology, hybrid nanoparticles derived from multiple cell types make precise targeting possible.^[^ ^9^, ^10^ ^]^ Neuroinflammation has been increasingly recognized as a critical factor in the pathogenesis of various NDs.^[^ ^11^, ^12^ ^]^ The activation of glial cells, including microglia and astrocytes, is a central feature of neuroinflammation in the CNS. These glial cells can produce and release a variety of proinflammatory cytokines, chemokines, and reactive oxygen species, which can cause damage to neurons and contribute to the pathogenesis of NDs.^[^ ^13^ ^]^ Among these mediators, chemokine (C–C motif) ligand 2 (CCL2), which predominantly originates from astrocytic sources, is important.^[^ ^14^ ^]^ CCL2 has been found to be upregulated in response to various neurological insults.^[^ ^15^, ^16^, ^17^ ^]^ This cytokine effectively recruits cells expressing its cognate receptor, chemokine (C–C motif) receptor 2 (CCR2), to afflicted regions, thereby augmenting the potential for targeted intervention. Motivated by these insights, our current focus is on designing a bioengineered cell membrane characterized by CCR2, a configuration tailored to facilitate drug accumulation in inflammation‐related lesions. Alzheimer's disease (AD) is the most common neurodegenerative disease. Notably, neuroinflammation is among the foremost events in AD pathogenesis.^[^ ^18^, ^19^, ^20^ ^]^ CCL2 secretion is increased in AD patients and animal models.^[^ ^21^, ^22^ ^]^ Therefore, the use of membranes with high expression of CCR2 for precise targeting of early‐stage lesions is a novel strategy for the effective treatment of AD.

Intriguingly, concurrent pathological changes in AD, such as the emergence of amyloid plaques, tau pathology, and neuronal degeneration frequently cooccur with neuroinflammation, forging a reinforcing interplay. Considering the multifaceted nature of AD and lessons from past unsuccessful attempts to develop drugs for AD, a combination of medications targeting different mechanisms is more advantageous than a single‐drug approach.^[^ ^23^ ^]^ Nanocarriers provide an opportunity not only to optimize dosing regimens but also to improve the effectiveness of multidrug therapies. In AD, a decrease in autophagy leads to an inability to clear away harmful proteins, resulting in neuronal death and glial cell activation, and ultimately triggering the development of a complex neuroinflammatory environment.^[^ ^13^, ^24^ ^]^ Several studies have demonstrated that rapamycin is a potential small molecule for enhancing autophagy and reducing the burden of pathological proteins in AD.^[^ ^24^, ^25^ ^]^ However, its extremely low BBB pass rate limits its clinical application.^[^ ^26^ ^]^ In addition, to align the carrier's function with targeting inflammatory lesions, we considered adding another drug that can inhibit neuroinflammation to improve the extracellular environment. 1‐Trifluoromethoxyphenyl‐3‐(1‐propionylpiperidin‐4‐yl) urea (TPPU), an inhibitor of soluble epoxide hydrolase (sEH), has been reported to prevent glial cell reactivation and alleviate cognitive deficits in the brains of AD mice.^[^ ^27^ ^]^ These results suggest that TPPU may be a potent candidate for encapsulation.

**Figure 1:**
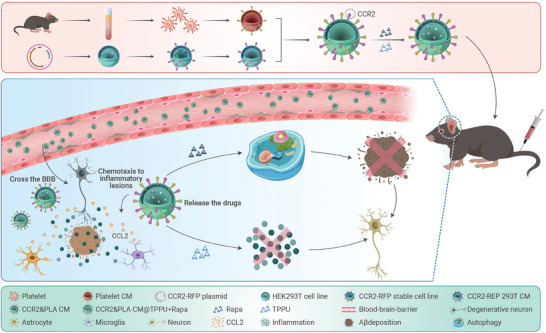
Scheme of the preparation of drug‐loaded hybrid cell membrane liposomes and their application for AD therapy. Platelet membranes were obtained from retro‐orbital blood collected from mice, and CCR2‐RFP overexpressing membranes were extracted from stable CCR2‐RFP‐transfected HEK293T cells. Two types of cell membranes were fused and loaded with rapamycin and TPPU. The drug‐loaded hybrid cell membrane liposomes migrated to inflammatory lesions in the CNS via intravenous injection. The drugs were released to enhance autophagy, alleviate neuroinflammation and ultimately treat AD.

Based on the above considerations, we proposed an original strategy that uses nanotechnology (**Figure** **1**). We developed a hybridization system by combining a platelet membrane and a membrane with high expression of CCR2, and loaded two small molecule drugs (rapamycin and TPPU) on these hybrid liposomes, to target and treat pathological areas in the brain associated with AD.

## 2 Results

### 2.1 CCR2‐RFP Is Located on the Cell Membrane and Mediates Chemotaxis toward CCL2

**Figure 2:**
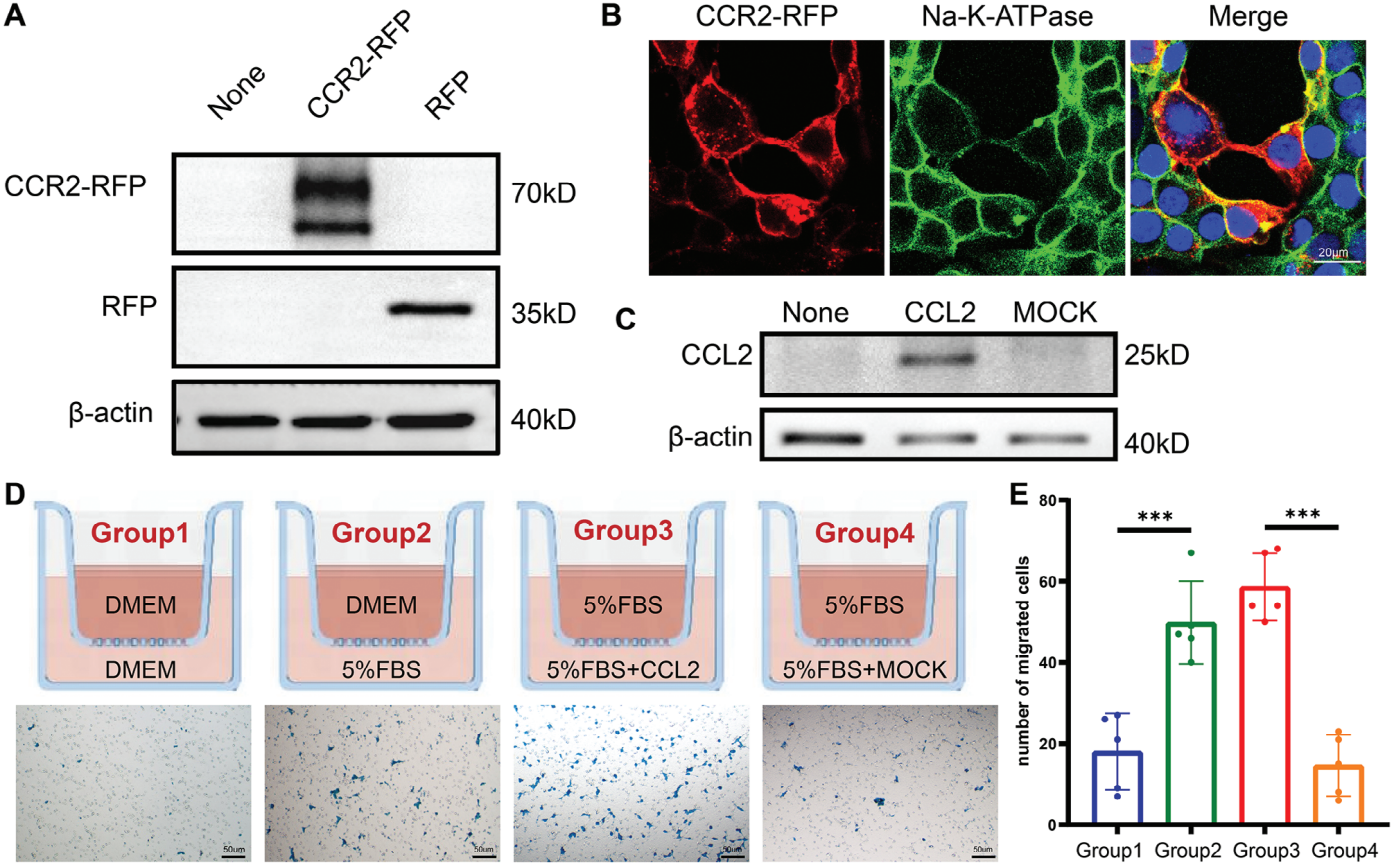
Expression, sublocalization and chemotaxis of CCR2‐RFP. A) Western blotting and B) immunofluorescence (scale bar: 20 µm) of HEK293T cells transfected with the CCR2‐RFP plasmid or mock vector. C) Western blotting of HEK293T cells transfected with the CCL2 plasmid. D) Schematic of the Transwell assay and representative images of migrated cells under different conditions (scale bar: 50 µm). E) Number of migrated cells in different groups (*n* = 5, ***p < 0.001).

In order to label the CCR2 protein, we fused it with an RFP (mScarlet). Firstly, we transfected the plasmid encoding the cDNA of the fusion protein into HEK293T cells. The CCR2‐RFP was found to be approximately 70 kDa (**Figure** **2**A) and located in the cell membrane (Figure 2B), which showed that RFP did not change the expression or cellular location of CCR2. To further verify whether CCR2 is expressed on the outer side of the cell membrane, we used flow cytometry to isolate cells with RFP, and found that approximately two‐thirds of these cells coexpressed CCR2 (Figure S1A, Supporting Information). This result showed that most of the CCR2 protein was located on the outer side of the cell membrane, and preliminarily indicated that it can function as a receptor.

Then, we wanted to clarify whether the fusion protein retained the chemotactic response of CCR2 to inflammatory cytokines. We constructed the plasmid of CCL2, and the overexpressed proteins were detected using Western blotting (Figure 2C). CCR2‐RFP and CCL2 were colocalized on the cell membrane after cotransfection (Figure S1B, Supporting Information), which may indicate a protein–protein interaction. We also used the Transwell assay to further test the migration of stable CCR2‐RFP transfected cells. As shown in Figure 2D,E, when the lower chamber contained more serum or CCL2, the number of migrated cells significantly increased. All these results demonstrate that CCR2‐RFP is located on the cell membrane and exhibits chemotaxis towards its ligand CCL2.

### 2.2 Preparation and Characterization of Hybrid Cell Membrane

**Figure 3:**
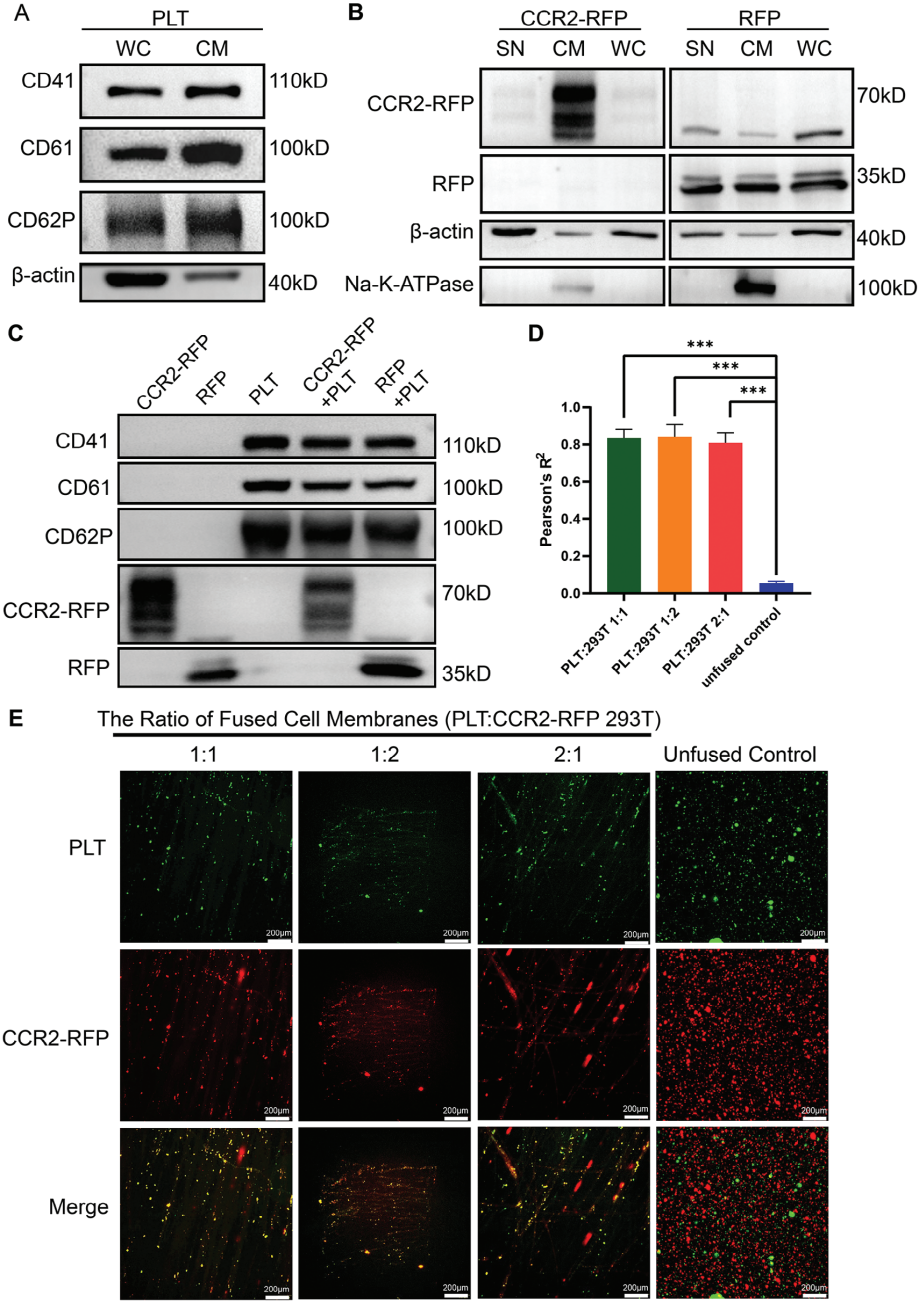
Extraction and hybridization of platelet membranes and CCR2‐RFP HEK293T cell membranes. A) Marker proteins (CD41, CD61, and CD62P) located in the membrane in different cell components of platelets. WC: whole cell; CM: cell membrane. B) Marker proteins (CCR2‐RFP and Na‐K‐ATPase) located in the membrane in different cell components of HEK293T cells. WC: whole cell; CM: cell membrane; SN: supernatant. C) Expression of marker proteins in the single cell membrane and hybrid cell membrane. D) Colocalization analysis of the hybrid cell membrane using Pearson's *R* ^2^ (*n* = 3, ***p < 0.001). E) Fluorescence images of colocalization in hybrid cell membranes with different fusion ratio (red: CCR2‐RFP HEK293T cell membrane; green: platelet cell membrane; scale bar: 200 µm).

We extracted the platelet membrane and overexpressed CCR2‐RFP HEK293T cell membrane separately. Coomassie brilliant blue staining revealed differences in the protein ladders among the different components (cell membrane, supernatant, and whole cell) of both cell membranes (Figure S2A,B, Supporting Information). We further blotted the marker proteins located on the membrane of the platelets (**Figure** **3**A) and CCR2‐RFP HEK293T cells (Figure 3B). There were three clusters of differentiation (CD) antigens (CD41, CD61 and CD62P) enriched in the cell membrane of platelets (Figure 3A). In HEK293T cells, the expression of CCR2‐RFP and Na‐K‐ATPase in the cell membrane was much greater than that of the other components. Moreover, RFP was broadly distributed in the cell membrane and cytoplasm (Figure 3B).

Then, we prepared cell membrane hybrid liposomes, and Western blotting demonstrated that the hybrid cell membrane coexpressed the marker proteins found in both platelets and HEK293T cells (Figure 3C). To verify whether these two kinds of cell membranes were successfully fused, we labeled them with different fluorophores. To determine the optimal fusion ratio, we also established three groups with different ratios of platelet and CCR2‐RFP HEK293T cell membranes (Group 1: PLT:293T = 1:1; Group 2: PLT:293T = 1:2; Group 3: PLT:293T = 2:1). We calculated the colocalization index (Pearson's *R* ^2^) based on fluorescence diagrams, and found that compared to those in the control group without extrusion, the cell membrane hybrid liposomes were strongly colocalized in all three groups at different ratios (Figure 3D,E). We successfully synthesized colocalized hybrid cell membranes that express characteristic membrane proteins.

### 2.3 Rapamycin and TPPU Rescue Cell Death in an In Vitro AD Model

It has been reported that autophagy and neuroinflammation play important roles in the occurrence and progression of AD. In 12 month old 5xFAD mice, the p‐mTOR/mTOR ratio was increased, while the LC3II/I ratio was decreased significantly (Figure S3A,C,D, Supporting Information), indicating that autophagy was inhibited in AD and exacerbated the burden of pathological proteins. In addition, soluble epoxide hydrolase (sEH), which is associated with a more severe inflammatory environment was found to be upregulated in AD mice (Figure S3B,E, Supporting Information). Therefore, we considered using an autophagy enhancer (rapamycin) and an sEH inhibitor (TPPU) to treat AD.

An in vitro AD model was subsequently established with ATRA and Aβ42(Figure S3F–H, Supporting Information). After treatment with rapamycin or TPPU for 24 h, we observed minimal drug toxicity at low concentrations. The optimal therapeutic concentrations of rapamycin and TPPU were found to be 250 and 1000 nmol L^−1^, respectively (Figure S3I–L, Supporting Information). These results show that rapamycin and TPPU can rescue cell death in an in vitro AD model, and we identified the appropriate concentration for treatment.

### 2.4 Preparation and characterization of Drug‐Loaded Hybrid Cell Membrane Liposomes

**Figure 4:**
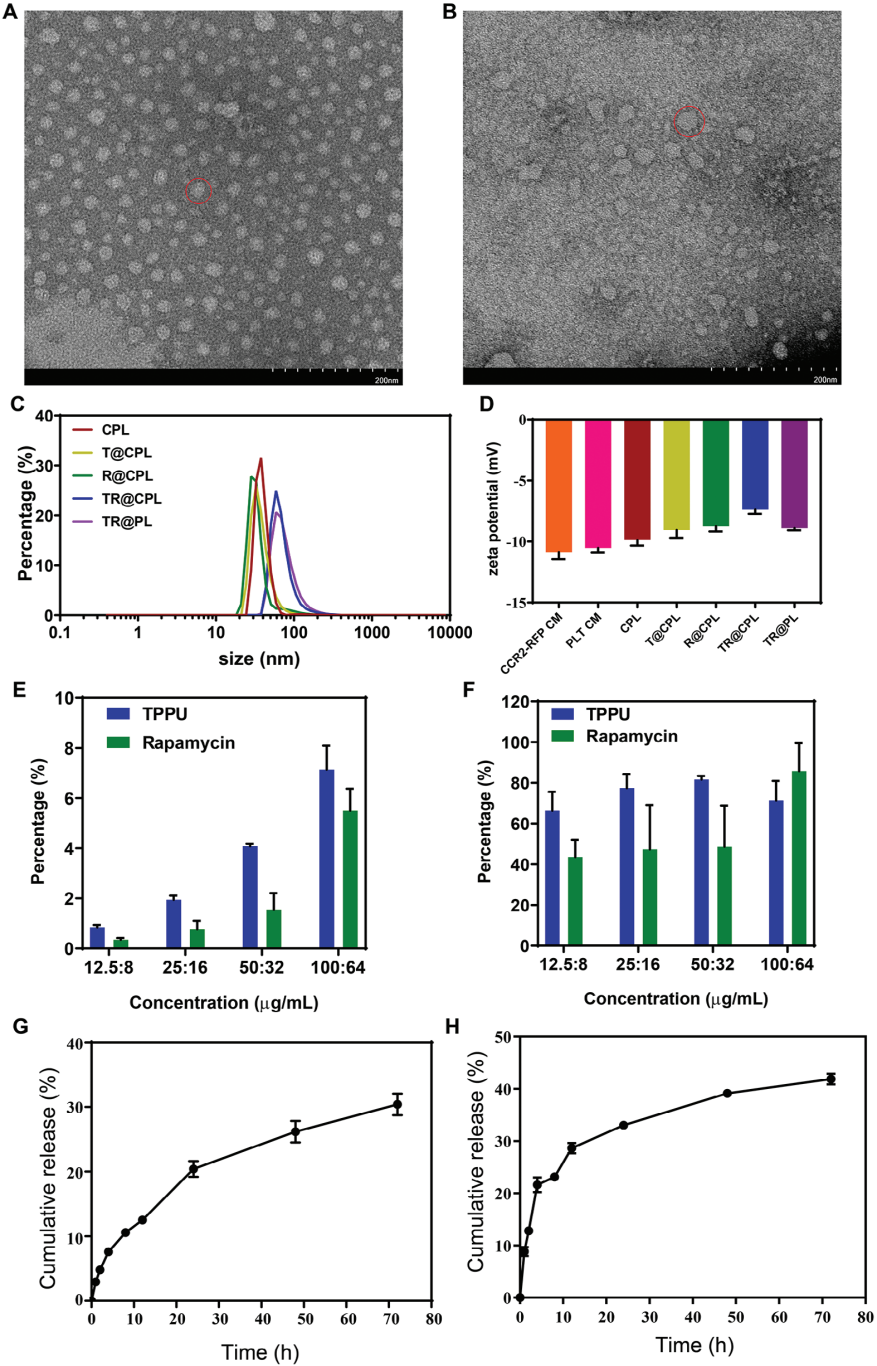
Characteristics of drug‐loaded hybrid cell membrane liposomes. TEM images of A) the hybrid cell membrane and B) the drug‐loaded hybrid cell membrane liposomes (scale bar: 20 nm). C) The particle sizes of different groups. D) The zeta potential of the different groups (*n* = 3). E) Loading efficiency and F) entrapment efficiency of cell membrane liposomes coloaded with rapamycin and TPPU (*n* = 3). Cumulative release of H) rapamycin and I) TPPU within 72 hours (*n* = 3).

The hybrid liposomes were observed via TEM (**Figure** **4**A,B). Regardless of whether the hybrid cell membrane liposomes contained drugs, they maintained a vesicle structure. However, the particle size of the hybrid liposomes loaded with two drugs (TR@CPL and TR@PL) was larger than that of the other liposomes (Figure 4C). The zeta potentials of the seven groups were roughly similar (Figure 4D). Then we analyzed the entrapment efficiency and loading efficiency of rapamycin and TPPU (Figure 4E,F; Figure S4, Supporting Information). We first tested the encapsulation of a single drug. The loading efficiency of 100 µg mL^−1^ TPPU had reached a stable level, but the entrapment efficiency decreased (Figure S4C, Supporting Information); rapamycin at a dose of 64 µg mL^−1^ exhibited the highest entrapment efficiency among all the tested groups (Figure S4D, Supporting Information). When rapamycin and TPPU were simultaneously added, the loading efficiency continued to increase (Figure 4E), and the optimal dose ratio of the two drugs was 100:64 µg mL^−1^ (TPPU: rapamycin) (Figure 4F). The cumulative release amounts of TPPU and rapamycin were approximately 30% and 40%, respectively, over 72 h (Figure 4G,H).

**Figure 5:**
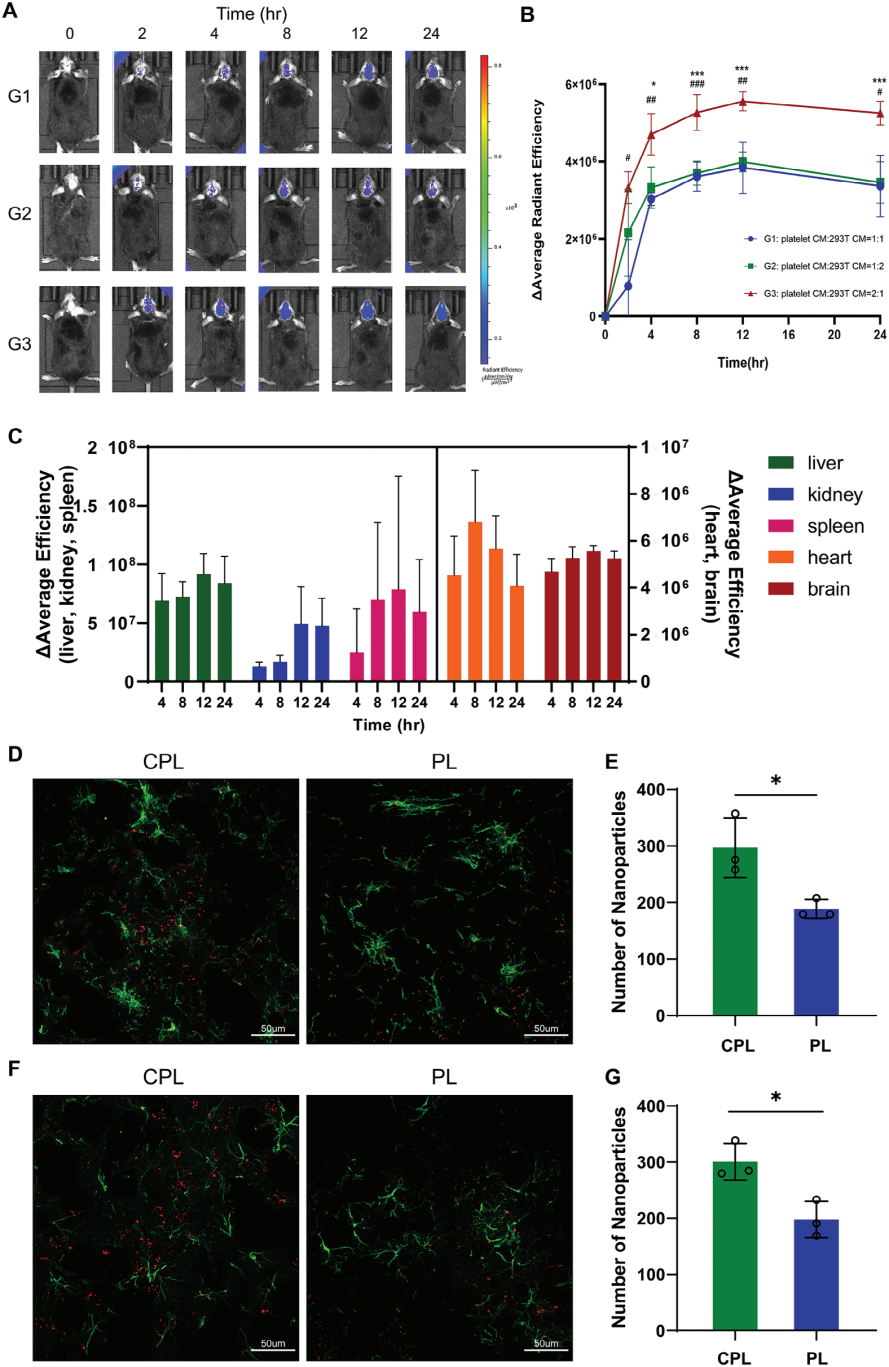
Biodistribution and targeting effect of hybrid cell membrane liposomes. A) Representative in vivo fluorescence images of 5xFAD mice treated with different ratios of hybrid cell membrane liposomes at various time‐points. B) Curve of the Δaverage radiant efficiency in the brain at various time‐points (*n* = 3, blue: G1, green: G2, red: G3; G1 versus G3: ^#^ *p* < 0.05, ^##^ *p* < 0.001, ^###^ *p* < 0.001; G2 versus G3: *p < 0.05, ***p < 0.001). C) Fluorescence biodistribution of different organs in G3 at various time‐points (*n* = 3). D) Immunofluorescence showing the migration of liposomes to microglia (green: Iba‐1; red: liposomes; scale bar: 50 µm). E) Number of liposomes around the microglia (*n* = 3, *p < 0.05). F) Immunofluorescence showing the migration of liposomes to astrocytes (green: GFAP; red: liposomes; scale bar: 50 µm). G) Number of liposomes around the astrocytes (*n* = 3, *p < 0.05).

To validate whether the hybrid liposomes can cross the BBB and migrate to inflammatory lesions in vivo, 5xFAD mice at 3 months of age were injected with DiR‐labeled liposomes. The fluorescence could be detected in the brain, and the signal reached its peak at 12 h (**Figure** **5**A). Comparing the three groups with different ratios of platelet and CCR2‐RFP HEK293T cell membranes, the pass rate of Group 3 (platelet CM: HEK293T CM = 2:1) was significantly greater than that of the other groups (Figure 5B). In addition to being enriched in the brain, liposomes were also enriched in the liver, spleen, and kidney (Figure 5C). We also found that liposomes could enter the CNS in wild‐type (WT) mice (Figure S5A, Supporting Information), but there was no significant difference among the three groups (Figure S5B, Supporting Information). In addition, more liposomes crossed the BBB in AD mice (Figure S5C, Supporting Information), which suggested the BBB dysfunction in AD. Twelve hours after intravenous injection, we extracted brain tissues from 5xFAD mice and prepared brain sections. Compared to the brain tissue of the group injected with the RFP HEK293T‐platelet hybrid membrane, the brain tissue of the group injected with CCR2‐RFP HEK293T‐platelet membrane hybrid liposomes showed liposome accumulation around microglia (Figure 5D,E) and astrocytes (Figure 5F,G). This indicates that the CPLs were able to target inflammatory lesions.

### 2.5 Drug‐Loaded Hybrid Cell Membrane Liposomes Ameliorate AD Symptoms Both In Vitro and In Vivo

First, we evaluated the biosafety and therapeutic efficacy of the liposomes in vitro. There were eight groups: PBS, TPPU, Rapamycin, TPPU+Rapamycin, T@CPL, R@CPL, TR@CPL, and TR@PL. According to the rescuing efficiency, the concentrations of rapamycin and TPPU were 2.3 and 3.6 µg mL^−1^, respectively. To comprehensively evaluate the role of liposomes in vitro, we selected various cell lines, including neuronal cells (HT22 hippocampal neuronal cell line, and SH‐SY5Y neuroblastoma cell line) and glial cells (BV2 microglial cell line). In the BV2 and HT22 cell lines, cell viability did not significantly decrease in any of the groups (Figure S6A,B, Supporting Information), which showed that the micromolecular drugs and liposomes had low biological toxicity. We further analyzed the therapeutic efficacy of SH‐SY5Y cells stably transfected with mutant APP (K595N/M596L), and BV2 and HT22 cells stimulated with Aβ42. The drugs and liposomes ameliorated the cell death caused by excessive Aβ42 (Figure S6C–E, Supporting Information). We also found that TR@CPLs performed better than single drug‐loaded liposomes and TR@PLs. Compared to uncoated drugs, CCR2‐RFP‐coated HEK293T and platelet cell membranes significantly increased the viability of BV2 cells (Figure S6D, Supporting Information).

**Figure 6:**
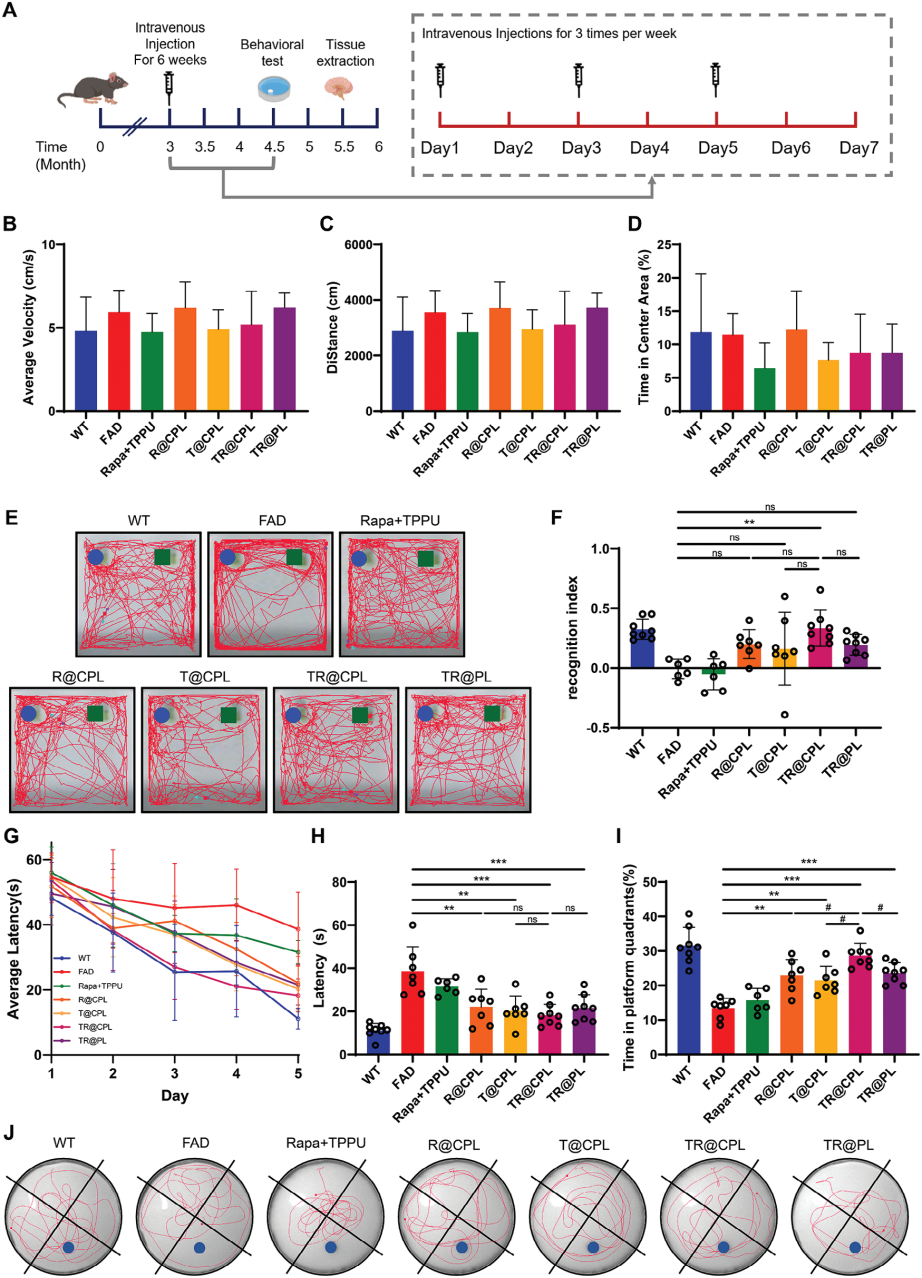
Behavioral tests of 5xFAD mice treated with different liposomes. A) Timeline of the animal experiment. B) Average velocity, C) distance and D) time spent in the center area by 5xFAD mice in the open field test (*n* = 6–8). E) Representative tracks of mice from seven groups in the NOR test. F) Recognition indices of mice in the NOR test (*n* = 6–8, *p < 0.05, ***p < 0.001, ns *p* > 0.05). G) Curve of average latency during the acquisition phase. H) Average latency of mice on Day 5 (*n* = 6‐8, **p < 0.01, ***p < 0.001, ns *p* > 0.05). I) Time spent in the platform quadrant (%) by the mice in the probe trial. J) Representative tracks of the mice in the probe trial. (*n* = 6–8, FAD versus liposome treatment: **p < 0.01, ***p < 0.001; TR@CPL versus the other three groups with liposome treatment: ^#^ *p* < 0.05).

The treatment of 5xFAD mice started at the age of 3 months via intravenous tail vein injections three times a week. After a 6 week treatment, we conducted behavioral tests on the mice. Then, we extracted tissues for subsequent experiments (**Figure** **6**A). We established seven groups as follows: 1) WT mice; 2) 5xFAD mice treated with vehicle; 3) 5xFAD mice treated with TPPU+rapamycin; 4) 5xFAD mice treated with R@CPL; 5) 5xFAD mice treated with T@CPL; 6) 5xFAD mice treated with TR@CPL; and 7) 5xFAD mice treated with TR@PL. The concentrations of rapamycin and TPPU in each group were 45.7 and 71.9 µg mL^−1^, achieving blood concentrations of 0.23 and 0.36 mg kg^−1^, respectively. The open field test is used to assess motor function, exploratory behavior, anxiety, and depression. There were no significant differences among the groups in terms of the velocity, moving distance or time spent in the center area (Figure 6B–D). We chose the NOR test to evaluate short‐term memory. Only the administration of TR@CPLs significantly improved cognition in 5xFAD mice (Figure 6E,F). For long‐term memory, as measured by the MWM test, the latency to reach the platform on training Day 5 was significantly shorter in the four groups treated with liposomes (Figure 6G,H). In the final test, we found that drug‐loaded hybrid cell membrane liposomes ameliorated cognitive deficiency (Figure 6I,J). Notably, the group treated with TR@CPLs were more effective than the other treatments (Figure 6I).

### 2.6 Drug‐Loaded Hybrid Cell Membrane Liposomes Reduced Amyloid Plaques and Alleviated Neuroinflammation

**Figure 7:**
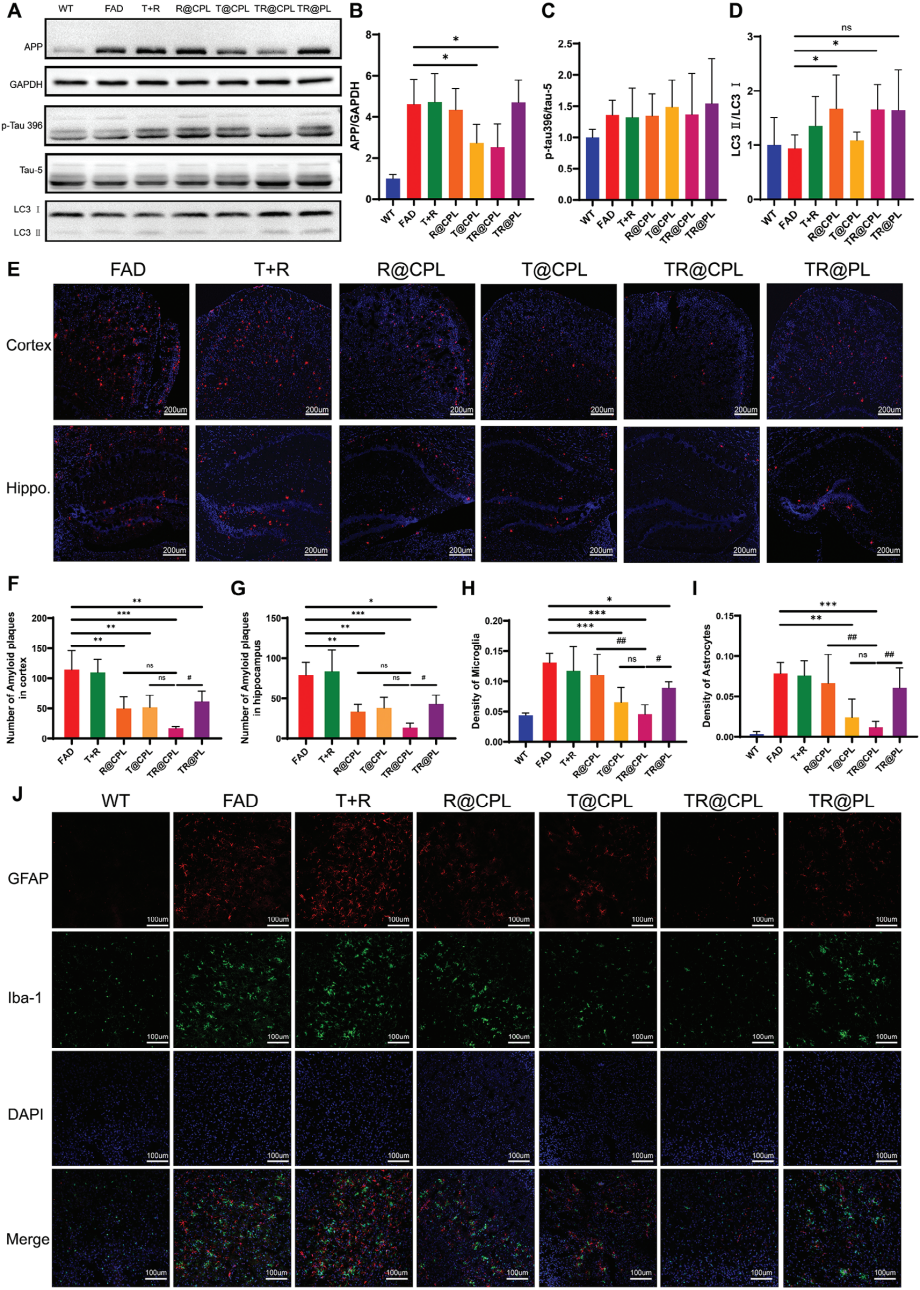
Drug‐loaded hybrid cell membrane liposomes reduced amyloid plaques and alleviated neuroinflammation. A) Western blot analysis of amyloid, tau, and autophagy related pathological proteins. Relative protein expression of B) APP, C) phosphorylated tau, D) LC3 (*n* = 5, FAD versus liposome treatment: *p < 0.05). E) Immunofluorescence of amyloid plaques in different treatment groups. The number of amyloid plaques in the F) cortex and G) hippocampus (*n* = 4‐5, FAD versus liposome treatment: *p < 0.05, **p < 0.01, ***p < 0.001; TR@CPL versus the other three groups with liposome treatment: ^#^ *p* < 0.05; scale bar: 200 µm). The density of H) microglia and I) astrocytes in the different treatment groups (*n* = 4‐5, FAD vs liposome treatment: *p < 0.05, **p < 0.01, ***p < 0.001; TR@CPL vs the other three groups with liposomes treatment: ^#^ *p* < 0.05, ^##^ *p* < 0.05). J) Immunofluorescence of glial cells in different treatment groups (scale bar: 100 µm).

After the behavioral tests, we carried out a series of pathological tests on the brain tissue. The expression of APP was significantly reduced in the T@CPL and TR@CPL groups (**Figure** **7**A,B), but there was no significant change in tau pathology (Figure 7A,C). Because the target of rapamycin is autophagy, we also detected the marker LC3II/I and found that its expression increased in the R@CPL and TR@CPL groups (Figure 7A,D), which indicated that rapamycin promoted autophagy.

Next, we observed amyloid plaques using immunofluorescence. All four groups with liposomes had moderate pathological protein burdens in both the cortex and hippocampus, with TR@CPL showing the most significant therapeutic effect (Figure 7E–G). Additionally, we wondered whether liposomes could alleviate neuroinflammation as reflected by glial cells. There was less glial cell infiltration in the brains in the T@CPL, TR@CPL, and TR@PL groups (Figure 7H–J). Moreover, compared to TP@PLs, TP@CPLs significantly reduced the number of glial cell aggregates (Figure 7H,I). This result suggested that TPPU has potential anti‐inflammatory effects, especially when combined with CCR2 overexpressing cell membrane coatings. Furthermore, we validated the biosafety of drug‐loaded hybrid cell membrane liposomes via HE staining. There were no apparent abnormalities in the heart, liver, spleen, lung, or kidney among the groups (Figure S7, Supporting Information).

## 3 Discussion

In this study, we innovatively designed a hybrid liposomal system enveloped in cell membrane coatings to treat AD. Our investigation was focused on two primary objectives. Initially, we elucidated the potential of this novel nanocarrier for precisely targeting inflammatory lesions within the brain through efficient BBB traversal. Subsequently, our research successfully encapsulated two separate therapeutic agents, rapamycin and TPPU, within hybrid cell membrane‐coated liposomes, thereby facilitating a synergistic and multitargeted therapeutic strategy against AD. The results from both in vitro and in vivo experiments have conclusively demonstrated the remarkable therapeutic potential of drug‐loaded hybrid cell‐membrane liposomes in the treatment of AD. Our findings indicate that the hybrid cell‐membrane‐coated liposome system has the potential to develop effective, precise and multitargeted treatments for AD. This opens the door to more effective treatment regimens and the potential for more personalized therapeutic strategies.

In recent years, the development of nanomaterials has revolutionized the field of medicine, particularly in the treatment of CNS diseases.^[^ ^28^ ^]^ Among the various nanotechnologies, cell membrane‐coated nanoparticles (CM‐NPs) have emerged as a promising approach for targeted therapy. CM‐NPs combine the benefits of both synthetic nanoparticles and natural cell membranes, offering enhanced biocompatibility, prolonged circulation time, and improved targeting ability.^[^ ^29^ ^]^ In the context of AD, these nanoparticles have shown great potential in overcoming BBB impermeability and delivering therapeutic agents specifically to affected brain regions.^[^ ^30^ ^]^ Among the cell membranes, the erythrocyte membrane is the most frequently used in AD therapy.^[^ ^31^, ^32^, ^33^ ^]^ Recent studies have also designed cell membrane camouflage nanosystems from microglial BV2 cells^[^ ^34^ ^]^ and macrophages^[^ ^35^ ^]^ against AD.

There is still a lack of therapeutic strategies for AD using platelet membranes as coatings. Theoretically, the platelet membrane has considerable advantages as a coating in the application of AD treatment. First, the platelet membrane expresses various membrane proteins. The presence of CD47 on the platelet membrane surface enables it to interact with signaling regulatory proteins on immune cells and inhibit immune cell‐mediated clearance of CM‐NPs,^[^ ^36^ ^]^ thereby prolonging the circulation of nanomedicine. Platelets also express unique surface ligands for targeting tissues or cells, especially damaged vascular systems. In the damaged vascular system, glycoprotein Ib/V/IX and von Willebrand factor bind to exposed collagen to form complexes, and platelets can initiate the coagulation process by binding to these complexes through integrins.^[^ ^37^ ^]^ Our results demonstrated that multiple integrins are expressed on the surface of mouse platelet membranes and can migrate around damaged blood vessels. The destruction of the BBB in AD is essentially a type of microvascular injury, which suggests that platelet membranes can pass through the BBB. As shown by the biodistribution analysis, cell‐membrane hybrid liposomes with a higher proportion of platelet membranes had a greater BBB penetration rate.

In addition to single cell membrane coated nanotechnology, several strategies have been employed to coat synthetic nanoparticles with cell membranes derived from different cell types, such as erythrocytes, neural stem cells, and macrophages. These hybrid nanoparticles can effectively mimic the surface properties and functionalities of source cells, enabling them to selectively interact with target cells or tissues.^[^ ^9^, ^10^ ^]^ However, research on the use of hybrid cell membrane‐coated nanoparticles for the treatment of neurological disorders (NDs) is still in its early stages. To date, kinds of hemocytes have been hybridized with cancer cells and applied to brain tumors.^[^ ^38^, ^39^, ^40^, ^41^ ^]^ However, there are no relevant reports on its application to other NDs. Furthermore, these nanodelivery systems are designed with wild‐type glial cell lines (C6 cell line, U251 cell line etc.) and are applied to target tumors through an isotypic targeting mechanism. Due to differences in pathological environments, tailoring characteristic biofilms through biological modification may be an important research subject for CM‐NPs.

Advances in cell isolation techniques and engineering approaches have facilitated the development of more sophisticated cell membrane‐coated nanomaterials.^[^ ^42^ ^]^ These advancements allow for the incorporation of additional factors with specific functionalities, such as specific ligands, antibodies, or imaging agents, onto the nanoparticle surface. This further enhances their targeting precision, therapeutic efficacy, and diagnostic capabilities. Here, we constructed a bioengineered cell membrane that overexpressed CCR2 and achieved inflammatory targeting in vivo. Although autologous microglia, astrocytes and neurons can express CCR2 on the membrane,^[^ ^43^ ^]^ our strategy may have several advantages. Compared to that of primary cells, the reproducibility of such engineered cells is superior. In addition, the expression abundance of the target protein can be greatly increased by lentivirus transfection. And another point is that primary cells are more sensitive and changeable. For example, there are two phenotypes of microglia, M1 and M2. At the same time, the two phenotypes can transform into each other under certain conditions.^[^ ^44^ ^]^ Microglia in the M1 state can promote the progression of disease,^[^ ^45^, ^46^ ^]^ while in the M2 state they function against neuroinflammation.^[^ ^47^ ^]^ However, it is difficult to distinguish and retain beneficial cell‐membranes in vitro, which makes this strategy difficult.

Nevertheless, despite the substantial progress made, challenges remain. The scalability and reproducibility of cell‐membrane coating methods need to be improved to meet clinical demands. The engineered extraction of extracellular vesicles (EVs) secreted from cells may provide some insights. A recent study revealed that microglial macrosomes reduce the amyloid burden and ameliorate AD, but microsomes have completely opposite effects.^[^ ^48^ ^]^ It is important to note whether the original cell membranes or EVs are damaged during preparation. Furthermore, the long‐term safety and potential immunogenicity of these nanomaterials must be thoroughly evaluated.

In addition to the nanocarrier, the encapsulated component is also important. For a long time, AD has been regarded as the most difficult “fortress” in the pharmaceutical research.^[^ ^49^ ^]^ At present, the first‐line drugs used to treat AD mainly include acetylcholinesterase inhibitors (donepezil)^[^ ^50^ ^]^ and glutamate receptor antagonists (memantine).^[^ ^51^ ^]^ Such drugs can improve cognition in early AD patients, but cannot cure the disease. Over the past few years, the research and development of single drug treatments for AD have repeatedly failed. These findings indicate that the therapeutic efficacy of single drug treatments is poor. Here, we loaded two different drugs into hybrid cell membrane‐coated liposomes for multitargeted therapy against AD. The use of multiple drugs with distinct mechanisms of action synergistic effects and addresses the multifaceted nature of AD pathology. For instance, one drug may target the accumulation of amyloid plaques, while the other may focus on reducing neuroinflammation or promoting neuronal survival. Our results indicate that 5xFAD mice benefit more from treatment with liposomes containing dual drugs (TR@CPLs) than single drugs (T@CPLs and R@CPL). This finding emphasizes the importance of a multitarget treatment protocol for AD. In addition, compatibility with appropriate carriers can improve the treatment efficacy of the drug. Compared to TR@PLs, which have a lower ability to target inflammation, TR@CPLs reduce the pathological protein burden much more and ameliorate neuroinflammation. Notably, both T@CPLs and TR@CPLs can decrease the expression of APP, which implies that the combination of TPPU and CPLs plays other roles in the pathological process of amyloid plaques.

Currently, various carrier substances have been developed for the treatment of AD. Disease‐modifying therapy for pathological protein burden has potential prospects.^[^ ^52^ ^]^ Recently, the tau‐targeting antisense oligonucleotide (ASO) MAPTRx has been reported to decrease tau protein and p‐tau protein levels in the cerebrospinal fluid.^[^ ^53^ ^]^ However, it is administered through intrathecal injection, implying that such biomacromolecules often have difficulty passing through the BBB. Diverse nanocarriers have been used to encapsulate peptides,^[^ ^54^ ^]^ plasmids,^[^ ^55^ ^]^ Cas9‐activating vectors,^[^ ^56^ ^]^ miRNAs^[^ ^57^ ^]^ or siRNAs^[^ ^58^ ^]^ for AD intervention. Since cell membrane nanocarriers provide a similar intracellular environment for proteins and nucleotides, the binding of these encapsulated substances to CM‐NPs may become a focus of future applications.

In conclusion, we prepared drug‐loaded hybrid cell‐membrane liposomes to achieve precise targeting of inflammatory lesions in the brain and multidrug treatment. This original nanomedicine can improve the cognition of AD model mice, reduce amyloid plaque deposition, and alleviate neuroinflammation. Hybrid cell membrane‐coated nanomaterials offer new opportunities for precise drug delivery and disease‐specific targeting, which represent a versatile platform for targeted therapy in AD. With continued advancements in nanotechnology and cell biology, hybrid cell membrane‐coated nanoparticles hold great promise as next‐generation therapeutic modalities for various neurological disorders.

## 4 Experimental Section

### Chemicals

Soybean phospholipid was purchased from Shanghai Yuanye Bio‐Technology Co., Ltd; cholesterol was purchased from Aladdin; and 1,2‐distearoyl‐sn‐glycero‐3‐phosphoethanolamine‐polyethylene glycol (DSPE‐PEG, *M*_w_ = 2000 Da) was purchased from Xi'an Ruixi Biological Technology Co., Ltd; Unless otherwise noted, organic solvents and inorganic salts were purchased from Sinopharm Chemical Reagent Co., Ltd., China, and used without further purification. Water in all the experiments was purified using a Millipore‐Q water purification system (Milli‐Q Integral 3, Millipore, USA).

### Animals

The 5xFAD transgenic mice generated via embryo transplantation were obtained from Model Organism (Shanghai, China). The transgenic mice coexpressed a total of five FAD mutations [APP K670N/M671L (Swedish) +I716V (Florida) + V717I (London) and PS1 M146L + L286V]. The mice were housed in groups of five, and maintained under standard laboratory conditions (20–22 °C, 65–70% relative humidity and a 12 h light/dark cycle). Food and water were available ad libitum throughout the experiments. The primers for genotyping were as follows: hAPP Forward primer: 5′AGAGTACCAACTTGCATGACTACG3’; hAPP Reverse primer: 5′ATGCTGGAT AACTGCCTTCTTATC3’; Internal Positive Control Forward primer: 5′CAAATG TTGCTTGTCTGGTG3’; Internal Positive Control Reverse primer: 5′GTCAGTCGA GTGCACAGTTT3’. All animal experiments followed the ethical regulations for drug research, training and experimentation promulgated by the Experimental Animal Ethics Committee of Zhejiang University and were carried out in accordance with the requirements of the National Act on the Use of Experimental Animals (People's Republic of China).

### Expression Plasmids

The full‐length wild‐type (WT) coding regions of musculus Ccr2 (ENSMUST 00 000055918.7) and Ccl2 cDNA (ENSMUST00000000193.6) were cloned and inserted into the PGMLV‐CMV‐MCS‐mScarlet‐PGK‐Puro and pIRES2‐EGFP vectors, respectively.

### Cell Culture and Plasmid Transfection

Stable CCR2‐red fluorescent protein (RFP) transfected HEK293T and RFP transfected HEK293T cells were generated by Genomeditech, China. All stably transfected cells were grown in Dulbecco's modified Eagle medium (DMEM) (Gibco, USA) supplemented with 10% fetal bovine serum (FBS) (Gibco, USA), 1% penicillin‒streptomycin (Gibco, USA) and 0.75 µg mL^−1^ puromycin (Beyotime, China) at 37 °C in 5% CO2. HEK293T, Neuro2a, HT22, SH‐SY5Y and BV2 cells were cultured at 37 °C under 5% CO2 in DMEM (Gibco, USA) supplemented with 10% FBS (Gibco, USA).

To simulate the pathology of AD in vitro, Neuro2a cells cultured in DMEM supplemented with 1% FBS were differentiated by 20 µmol L^−1^ all‐trans retinoic acid (ATRA) (Sangon Biotech, China) for 48 h. Subsequently, the cells were stimulated with 10 µmol L^−1^ Aβ42 peptide (Shanghai Maokang Biotechnology Co., China) for 24 h.

To determine the expression and subcellular location of proteins, HEK293T cells were transfected with plasmids with Lipofectamine 3000 reagent (Invitrogen, USA) according to the manufacturer's protocol. 48 hours after transfection, the transfected cells were collected for further analysis.

### Cell Counting Kit‐8 (CCK‐8) Assay

CCK‐8 (Beyotime, China) was used to measure cell proliferation. A total of 10^4^ cells in a volume of 100 µL per well were cultured in four replicate wells in a 96‐well plate. Then, the CCK‐8 reagent (10 µL) was added to 100 µL of DMEM to generate a working solution, of which 110 µL was added per well and incubated for 2–4 h. The absorbance was measured at 450 nm.

### Flow Cytometry

Plated cells were collected in cold PBS. Antibodies were diluted in 1% bovine serum albumin (BSA) (Sigma‐Aldrich, USA). A total of 10^6^ cells were incubated in 100 µL of antibody diluent on ice for 30 min in the dark. The following antibodies were used: FITC‐conjugated anti‐mouse CD192 (CCR2) (1:200, BioLegend, USA), FITC‐conjugated rat IgG2b, and κ Isotype Ctrl (1:200, BioLegend, USA). After incubation, the samples were centrifuged at 1000 rpm and the supernatant was discarded. The fluorescence intensity of the cells was detected via the BD FACSCanto II flow cytometer.

### Transwell Migration Assay

A total of 10^5^ stable CCR2‐RFP transfected HEK293T cells were plated in the upper chamber of a Transwell‐Clear Insert, polyester (PET) membrane (Corning, USA) with 200 µL of culture medium. The lower chamber contained cells transfected with the CCL2 plasmid or mock vector in 500 µL of culture medium. After 24 h of migration, the cells were fixed with methanol for 30 min and stained with crystal violet staining solution (Beyotime, China) for 5 min. Nonmigrated cells were removed with a cotton swab and migrated cells were counted under the microscope.

### Extraction of the Cell Membrane

The HEK293T cell membrane was extracted according to the protocol of the Membrane and Cytosol Protein Extraction Kit (Beyotime, China). Specifically, HEK293T cells were digested with 0.25% trypsin (Gibco, USA), and washed three times with cold PBS. The cells were lysed with cell membrane protein extraction solution A containing 1 mmol L^−1^ phenylmethanesulfonyl fluoride (PMSF) (Beyotime, China) for 15 min on ice. The samples were quickly frozen in liquid nitrogen and thawed at 37 °C for six repetitions. After centrifugation at 1000 *g* for 10 min, the gelatinous substances were discarded. The cell membrane was obtained by centrifugation at 14 000 *g* for 30 min and was dissolved in Milli‐Q water with 10% glycerol (Sangon Biotech, China).

Whole blood samples were collected from the inner canthal orbital vein of anesthetized mice and stored in 6 mL BD Vacutainer K2 EDTA tubes (Becton Dickinson, USA) with Anticoagulant ACD Solution (Macklin, China). Platelets were extracted by centrifugation at 100 *g* for 20 min and purified three times using red blood cell lysis buffer (Solarbio, China). The purified platelets were dissolved in Tyrode's solution (Solarbio, China) supplemented with 1 mmol L^−1^ PMSF (Beyotime, China). The samples were quickly frozen and thawed three times as per the protocol for HEK293T cell membrane extraction. After ultrasonication at 10% intensity for 5 mi, the platelet membrane was obtained by centrifugation at 12 000 rpm for 30 min and dissolved in Milli‐Q water with 10% glycerol (Sangon Biotech, China).

### Preparation and Characterization of Hybrid Liposomes

Liposomes were prepared by the thin lipid film hydration method.^[^ ^59^ ^]^ Briefly, 35 mg of soybean phospholipids, 5 mg of cholesterol, and 10 mg of DSPE‐PEG were dissolved in 10 mL of chloroform to obtain a 5 mg mL^−1^ mixed phospholipid solution. TPPU and Rapa were dissolved in ethanol to form 1 mg mL^−1^ stock solutions. Then, 1 mL of the 5 mg mL^−1^ mixed phospholipid solution, 0.5 mL of 1 mg mL^−1^ TPPU, 0.064 mL of the 1 mg mL^−1^ Rapa, and 10 mL of chloroform were added to a round flask and evaporated slowly to form a thin film by a rotary evaporator at 40 °C. Thereafter, sterile PBS (pH 7.4, 10 × 10^−3^ m) was added to the round flask and sonicated for 20 min in an ice water bath to obtain double drug‐loaded liposomes (TR@L). Single drug‐loaded liposomes were obtained using the same method, except that only TPPU or Rapa was added (T@L or R@L).

Cell membrane hybrid liposomes were obtained via an extruder set (Avanti Polar Lipids, Inc., USA). The liposomes and proteins from the cell membrane (1:1, w/w) were mixed and subsequently sonicated in an ice water bath for 5 min. Next, the mixtures were extruded in order ten times at 42 °C through 200, 100, and 50 nm polycarbonate porous membrane filters using an extruder. Finally, the free drugs were removed through ultrafiltration tubes (Millipore, MWCO = 3000 Da). Four types of hybrid liposomes were obtained: CCR2‐platelet membrane hybrid TR@L (TR@CPL), CCR2‐platelet membrane hybrid T@L (T@CPL), CCR2‐platelet membrane hybrid R@L (R@CPL), and HEK 293T‐platelet membrane hybrid TR@L (TR@PL).

The hydrodynamic diameter and zeta potential of all liposomes were determined by dynamic light scattering (DLS, Zetasizer 3000, Malvern, USA). The measurements were was repeated three times to obtain an average value. The morphology was observed under a transmission electron microscope (TEM, HT‐7700, Hitachi, Japan) at 200 kV after placing a drop of the liposome solution on a copper grid coated with amorphous carbon and stained with a phosphotungstic acid solution (2%).

### Drug Loading Capacity and Drug Release Manner of Hybrid Liposomes

To optimize the drug loading efficiency, the drug dose was adjusted during the preparation of the liposomes. After preparing the hybrid liposomes through an extruder, the unencapsulated free drugs were removed using ultrafiltration tubes. Then, the encapsulated drugs were collected by extracting the liposomes with ethanol and measured by an ultraviolet spectrometer (UV, UH5300, Hitachi, Japan). The standard curves of the drugs were obtained at the same time. The encapsulation efficiency was calculated as the percentage of encapsulated drug to the total drug dose. The loading efficiency was calculated as the percentage of encapsulated drug to the total liposome mass.

Then, the drug release from the liposomes was investigated. Specifically, 1 mL of TR@CPL was packed into a dialysis tubing (MWCO = 3500 Da) and immersed in 19 mL of PBS (pH 7.4, 10 × 10^−3^ m) in a 37 °C water bath with orbital shaking at 200 rpm. At predetermined time intervals, 0.5 mL of PBS was removed, followed by the addition of another 0.5 mL of fresh PBS. The amount of drug released was determined by UV spectroscopy.

### Biodistribution In Vivo

To evaluate BBB penetration and the distribution of hybrid cell membranes, and determine the optimal fusion ratio of the cell membrane, different ratios of DiR‐labeled hybrid cell membranes (1 mg kg^−1^) were injected into mice via the tail vein. At various time points, the mice were imaged using the IVIS Lumina III Imaging System (PerkinElmer, China). The Δaverage radiant efficiency in the region of interest was used to assess the enrichment of fluorescence.

(1) $$\begin{array}{ccc} & & \Delta \mathrm{Average}\;\mathrm{Radi}\mathrm{ant}\;\mathrm{Effi}\mathrm{cien}\mathrm{cy}\\ & & \;=\mathrm{Aver}\mathrm{age}\;\mathrm{Radi}\mathrm{ant}\;\mathrm{Efficiency}\left(X\;\mathrm{hr}|\;\mathrm{certain}\;\mathrm{mouse}\right)\\ & & \;-\;\mathrm{Aver}\mathrm{age}\;\mathrm{Radi}\mathrm{ant}\;\mathrm{Effi}\mathrm{cien}\mathrm{cy}\;\left(0\;\mathrm{hr}|\mathrm{certain}\;\mathrm{mouse}\right) \end{array}$$

### Tissue Extraction and Sectioning

Mice were anesthetized with 0.3% pentobarbital and sacrificed by heart perfusion with PBS. Then, the brain was rapidly dissected on ice and half of the brain tissue was separated into the cortex and hippocampus, and stored at −80 °C for further analysis. The other half of the brain tissue was fixed in 4% PFA (Sangon Biotech, China) for 24 h and dehydrated with 30% sucrose (Sangon Biotech, China) in PBS. In addition, we collected other organs, including the heart, lung, spleen, and kidney, and fixed them in 4% PFA. Tissue sections were prepared with optimal cutting temperature (OCT) compound (Sakura, USA) embedding and a freezing microtome (Leica, Germany).

### Western Blotting and Coomassie Brilliant Blue Staining

Mouse brain tissues and cultured cells were homogenized and lysed in the RIPA lysis buffer (Beyotime, China) supplemented with 1% protease inhibitor PMSF (Beyotime, China), and phosphatase inhibitors (MCE, China). Then the lysis solutions were centrifuged at 12 000 rpm for 30 min at 4 °C. Protein extracts were separated on SDS‐PAGE gels and transferred to polyvinylidene fluoride membranes. The following primary antibodies were used: RFP antibody (1:1000, ChromoTek, Germany), α1‐sodium potassium ATPase antibody (1:1000, Abcam, USA), MCP1 antibody (1:1000, Abcam, USA), integrin β3 (CD61) antibody (1:1000, CST, USA), CD41 antibody (1:1000, Abcam, USA), CD62P antibody (1:1000, ABclonal, China), APP antibody (1:1000, CST, USA), p‐mTOR antibody (1:1000, CST, USA), mTOR antibody (1:1000, CST, USA), EPHX2 antibody (1:1000, ABclonal, China), LC3 II/I antibody (1:1000, CST, USA), Flag antibody (1:5000, ABclonal, China), GAPDH‐HRP antibody (1:10000, ABclonal, China) and β‐actin‐HRP antibody (1:10000, HuaBio, China). HRP‐conjugated secondary antibodies were used to detect the primary antibodies.

Coomassie brilliant blue staining used Coomassie Blue Staining Kit (Beyotime, China). After electrophoresis, the SDS‐PAGE was incubated with Coomassie R250 dye for two hours, and then washed with the eluent for four hours at room temperature.

### Immunofluorescence Analysis

Brain sections and cells were fixed with 4% paraformaldehyde (PFA) (Sangon Biotech, China) for 5 min at room temperature and blocked in PBS containing 0.3% Triton X‐100 (Sangon Biotech, China) and 10% normal donkey serum (Jackson, USA) for 60 min. The samples were then incubated with primary antibodies in blocking buffer at 4 °C overnight, followed by incubation with fluorescence‐conjugated secondary antibodies for 1 h at room temperature. The following primary antibodies were used: α1‐sodium potassium ATPase antibody (1:100, Abcam, USA), Flag antibody (1:500, ABclonal, China), β‐Amyloid antibody (1:500, CST, USA), Iba‐1 antibody (1:200, Oasis, China) and GFAP antibody (1:500, Abcam, USA). The cell nuclei were then stained with 40, 6‐diamidino‐phenylindole (DAPI) (1:10 000, Life Technologies, USA). Fluorescence images were acquired with a Zeiss 900 confocal system.

### Hematoxylin‐Eosin (HE) Staining

The Hematoxylin and Eosin Staining Kit (Beyotime, China) was used for histopathological examination. Sections were fixed with 4% PFA for 10 min, followed by hematoxylin staining for 10 mi and eosin staining for 2 mi. After gradient dehydration with ethanol and clarification with dimethylbenzene, the sections were observed under a light microscope.

### Behavioral Test

4.5 month old mice treated with different types of drug‐loaded cell membrane hybrid liposomes were evaluated for each behavioral tests, including the open field test, novel object recognition test and the Morris water maze. Prior to testing, all the animals were handled for five consecutive days for 10 mi each day, and were allowed to habituate to the test environment for 30 min. Behavioral tests were performed at the behavioral test core of the School of Medicine, Hangzhou City University and analyzed with an automated system.

### Open Field Test

Each mouse was placed in the center of a transparent plastic chamber (40 × 40 × 40.5 cm) and allowed to explore freely for 10 min. The testing arena was brightly lit. During each session, their behavior was automatically videotaped and subsequently analyzed using the EthoVision video tracking system.

### Novel Object Recognition (NOR)

During the test phase, the mice were initially placed individually in the center of the open field. Two copies of an object were located in ipsilateral corners, seven ceilometers from the walls of the open field. Ten minutes of exploration were recorded in this sample trial. After a 2 h delay, the mice underwent a second 10 min probe test trial where a copy of the original or familiar object and a second novel object were used. Their behavior was automatically videotaped and subsequently analyzed using the EthoVision video tracking system.

(2) $$\begin{array}{ccc} & & \mathrm{Recognition}\;\mathrm{index}\;\\ & & \;=\left(\mathrm{exploration}\;\mathrm{time}\;\mathrm{of}\;\mathrm{novel}\;\mathrm{object}-\mathrm{exploration}\;\mathrm{time}\;\mathrm{of}\;\mathrm{original}\right.\\ & & \;\left.\mathrm{object}\right)/\;\left(\mathrm{exploration}\;\mathrm{time}\;\mathrm{of}\;\mathrm{novel}\;\mathrm{object}\;+\;\mathrm{exploration}\;\mathrm{time}\right.\\ & & \;\left.\mathrm{of}\;\mathrm{original}\;\mathrm{object}\right) \end{array}$$

### Morris Water Maze (MWM)

The Morris water maze was conducted according to the protocol.^[^ ^60^ ^]^ The spatial acquisition phase consisted of four training trials per day over 5 training days with an intertrial interval of 20 min. Mice were released randomly from the four compass locations, and allowed to swim and search for the platform for one minute. If the mice did not find the platform within one minute, they were manually placed on the platform and allowed to remain on it for 10 s.

One day after the acquisition phase, the mice underwent a probe trial, in which the platform was removed. Mice were released from the northeast starting point and allowed to swim freely for one minute. Paths were traced and analyzed to determine the proportion of swim time spent in the platform quadrant. Their behavior was automatically videotaped and subsequently analyzed using the EthoVision video tracking system.

### Statistical Analysis

All the data were obtained from at least three independent experiments, and the experimental data are presented as the mean ± standard error of the mean (SEM). All the statistical analyses were performed with GraphPad Prism 7 software. Student's t‐test was used for comparisons between two groups. Statistical analysis of multiple groups used One‐way ANOVA. Pairwise comparisons were performed with Dunnett's multiple comparisons test. Significant differences were strictly accepted when *p < 0.05 and **p < 0.01 or ***p < 0.001.

### Ethics Approval Statement

The study was approved by the Ethics Committee of Second Affiliated Hospital, Zhejiang University School of Medicine (No.2023115).

## Conflict of Interest

The authors declare no conflict of interest.

## Author Contributors

R.‐R.L. and L.‐L.J. contributed equally to this work. R.‐R.L.: data collection, analysis and interpretation of the data, and drafting the manuscript; L.‐L.J.: data collection, analysis and interpretation of the data; Y.‐Y.X.: data collection, analysis and interpretation of the data; Z.‐S.Z.: data collection, analysis and interpretation of the data; H.‐F.H.: data collection; D.‐F.C.: data collection; Q.L.: data collection; Z.‐W.M.: designed the study, analysis and interpretation of the data, critical revision of the manuscript; Z.‐Y.W.: funding, conceptualized and designed the study, critical revision of the manuscript; Q.‐Q.T.: funding, conceptualized and designed the study, critical revision of the manuscript. All authors approved the final manuscript.

## Supporting information

Supporting Information

## Data Availability

The data that support the findings of this study are available from the corresponding author upon reasonable request.
