# Supplementary material for: Hybrid Membrane‐Coated Nanoparticles for Precise Targeting and Synergistic Therapy in Alzheimer's Disease
Source: Adv Sci (Weinh). 2024 Apr 22;11(24):2306675. doi: 10.1002/advs.202306675 (PMC11200089; doi:10.1002/advs.202306675)
Supplement: Supplementary file 1 — Supporting Information [file ADVS-11-2306675-s001.pdf]

ADVANCED SCIENCE
Open Access

Supporting Information

for *Adv. Sci.*, DOI 10.1002/advs.202306675

Hybrid Membrane-Coated Nanoparticles for Precise Targeting and Synergistic Therapy in Alzheimer's Disease

Rong-Rong Lin, Lu-Lu Jin, Yan-Yan Xue, Zhe-Sheng Zhang, Hui-Feng Huang, Dian-Fu Chen, Qian Liu, Zheng-Wei Mao, Zhi-Ying Wu* and Qing-Qing Tao**

Supplementary Figures

Figure S1. Both CCR2-RFP and CCL2 are located on the cell membrane. (A) Flow cytometry of HEK293T cells transfected with CCR2-RFP. (B) Immunofluorescence of HEK293T cotransfected with the CCR2-RFP plasmid and CCL2 plasmid (Scale bar: 20 μm).

Figure S2. SDS-PAGE gels of Coomassie brilliant blue staining showed different components from (A) CCR2-RFP HEK293T cells and (B) platelets.

Figure S3. Dual-drug strategy based on AD pathology. Western blotting of proteins related to (A) autophagy and (B) inflammation in 5xFAD and WT mice (N=3 or 6). Relative expression of (C) mTOR signaling, (D) LC3 autophagosome and (E) sEH in 5xFAD and WT mice (N=3 or 6, *$p < 0.05$, ***$p < 0.001$). The morphology of (F) normal Neuro2a cells, (G) neuronal-like Neuro2a cells induced by ATRA, and (H) the AD model *in vitro* constructed by subsequently stimulating the ATRA-induced Neuro2a cells with Aβ42(N=3, ***$p < 0.001$). The (I) toxicity and (J) rescuing efficiency of rapamycin. The (K) toxicity and (L) rescuing efficiency of TPPU (N=3, *$p < 0.05$, ***$p < 0.001$).

Figure S4. Characteristics of drug-loaded hybrid cell membrane liposomes. Absorbance curve of (A) TPPU and (B) rapamycin. The entrapment and loading efficiency of (A) TPPU and (B) rapamycin.

Figure S5. Biodistribution of hybrid cell membrane liposomes in WT mice. (A) Representative *in vivo* fluorescence images of WT mice treated with different ratios of hybrid cell membrane liposomes at various time-points. (B) Curve of

the ΔAverage Radiant Efficiency in the brain at various time-points (N=3, blue: G1, green: G2, red: G3). (C) Curve of the ΔAverage Radiant Efficiency in the brain of G3 at various time-points (N=3, blue: 5xFAD mice, green: WT mice; $*p < 0.05$, $**p < 0.01$). (D) Fluorescence biodistribution of different organs at 12 hours (N=3, blue: G1, green: G2, red: G3).

Figure S6. The toxicity and efficiency of drug-load hybrid cell membrane liposomes *in vivo*. Viability (%) of (A) HT22, (B) BV2 and (C) mutant APP-expressed SH-SY5Y cells (N=3, PBS vs. treatment groups: $***p < 0.001$; $^{##} p < 0.001$, $^{###}p < 0.001$, ns $p > 0.05$). Viability (%) of (D) HT22 and (E) BV2 cells incubated with Aβ42 (N=3, Aβ42 vs. treatment groups: $**p < 0.01$, $***p < 0.001$; $^{#} p < 0.05$, $^{##} p < 0.001$, $^{###}p < 0.001$).

Figure S7. HE staining of different organs with treatment of drug-loaded hybrid cell membrane liposomes (Scale bar: 100 μm).

Figure S1

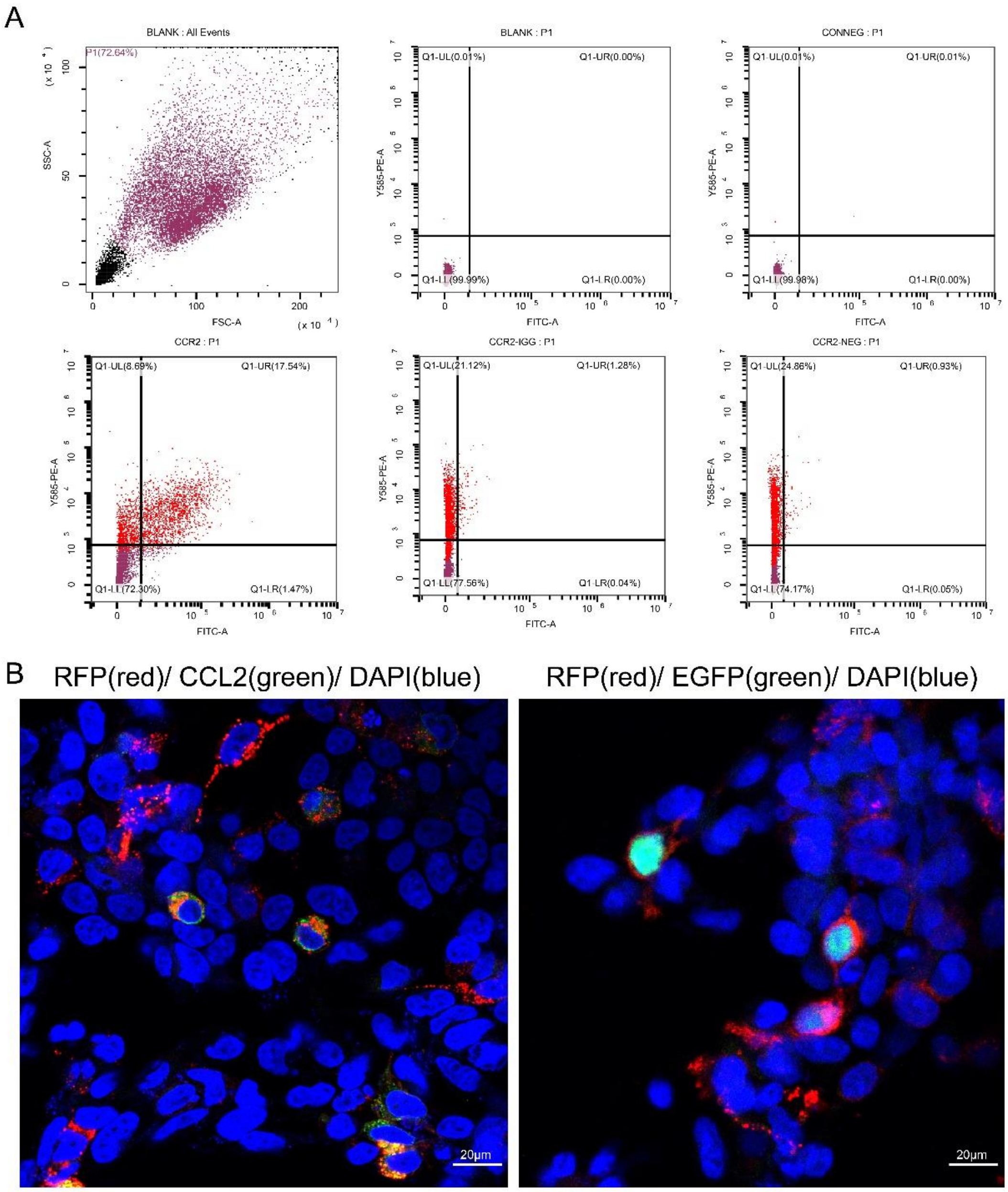
A
BLANK : All Events
P1(72.64%)
SSC-A
FSC-A
BLANK : P1
Q1-UL(0.01%)
Q1-UR(0.00%)
Q1-LL(99.99%)
Q1-LR(0.00%)
CONNEG : P1
Q1-UL(0.01%)
Q1-UR(0.01%)
Q1-LL(99.98%)
Q1-LR(0.00%)
CCR2 : P1
Q1-UL(8.69%)
Q1-UR(17.54%)
Q1-LL(72.30%)
Q1-LR(1.47%)
CCR2-IGG : P1
Q1-UL(21.12%)
Q1-UR(1.28%)
Q1-LL(77.56%)
Q1-LR(0.04%)
CCR2-NEG : P1
Q1-UL(24.86%)
Q1-UR(0.93%)
Q1-LL(74.17%)
Q1-LR(0.05%)
Y585-PE-A
FITC-A
B
RFP(red)/ CCL2(green)/ DAPI(blue)
RFP(red)/ EGFP(green)/ DAPI(blue)
20μm
20μm

Figure S2

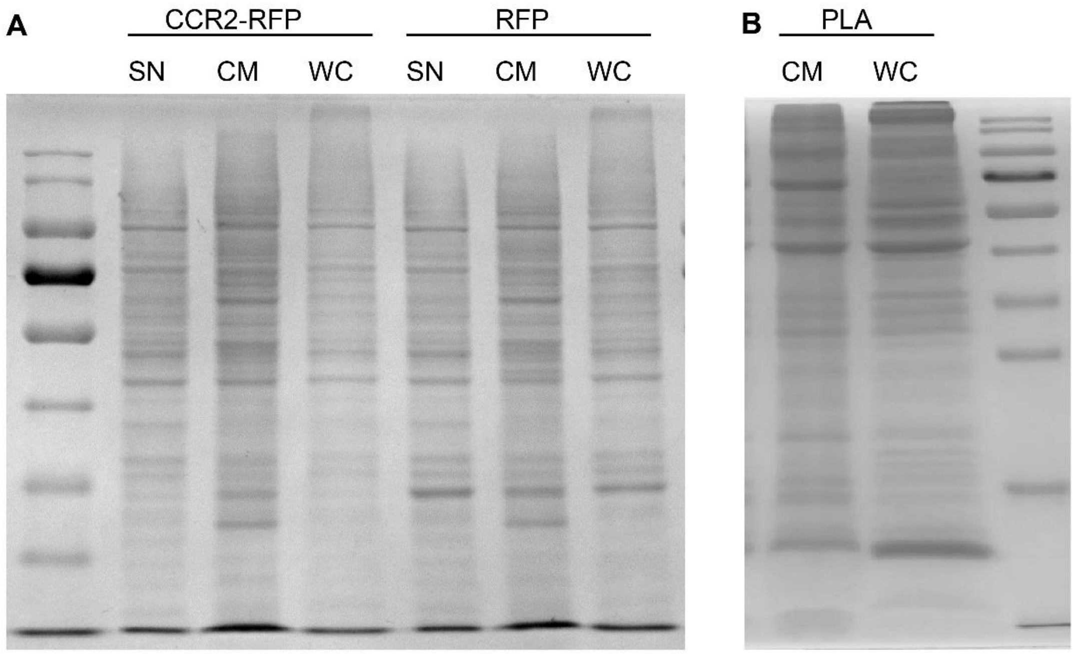

Figure S3

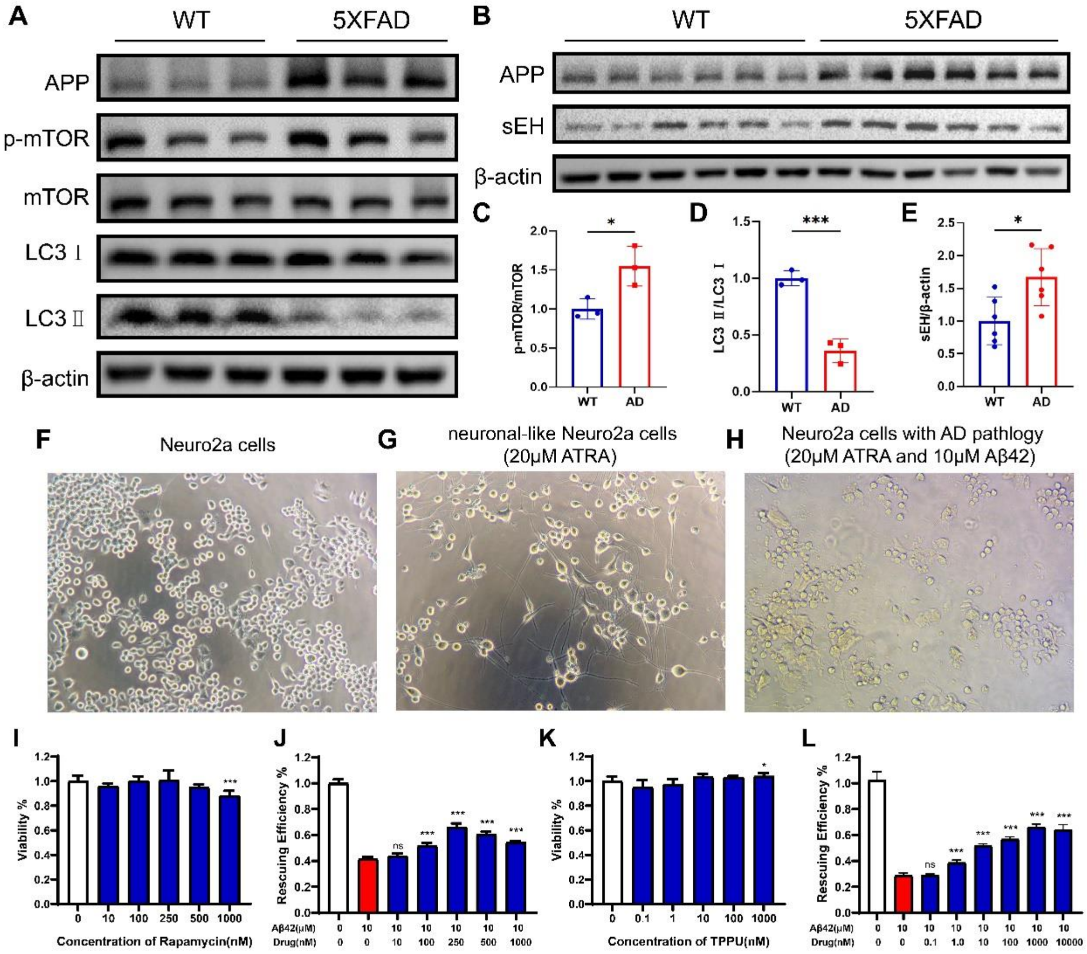

A
WT
5XFAD
APP
p-mTOR
mTOR
LC3 Ⅰ
LC3 Ⅱ
β-actin
B
WT
5XFAD
APP
sEH
β-actin
C
p-mTOR/mTOR
WT
AD
D
LC3 Ⅱ/LC3 Ⅰ
WT
AD
E
sEH/β-actin
WT
AD
F
Neuro2a cells
G
neuronal-like Neuro2a cells (20μM ATRA)
H
Neuro2a cells with AD pathlogy (20μM ATRA and 10μM Aβ42)
I
Viability %
Concentration of Rapamycin(nM)
J
Rescuing Efficiency %
Aβ42(μM)
Drug(nM)
K
Viability %
Concentration of TPPU(nM)
L
Rescuing Efficiency %
Aβ42(μM)
Drug(nM)

Figure S4

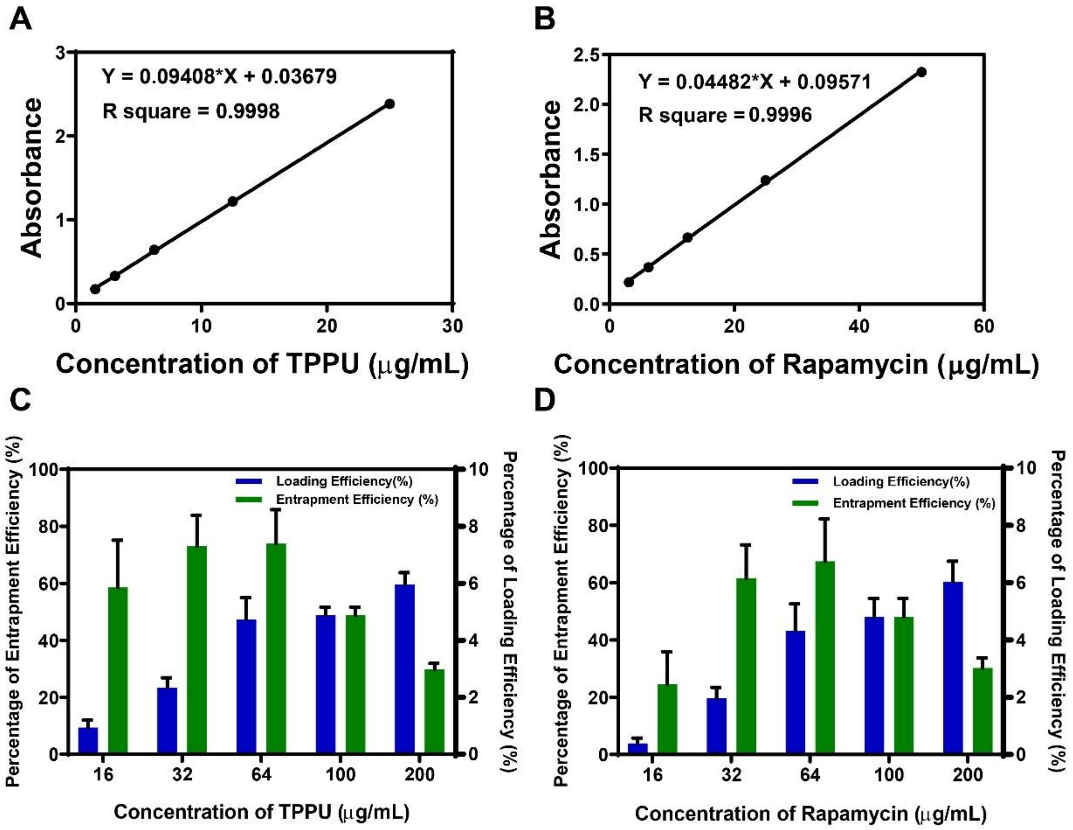
A
Y = 0.09408*X + 0.03679
R square = 0.9998
Absorbance
Concentration of TPPU (μg/mL)
B
Y = 0.04482*X + 0.09571
R square = 0.9996
Absorbance
Concentration of Rapamycin (μg/mL)
C
Loading Efficiency(%)
Entrapment Efficiency (%)
Percentage of Entrapment Efficiency (%)
Percentage of Loading Efficiency (%)
16
32
64
100
200
Concentration of TPPU (μg/mL)
D
Loading Efficiency(%)
Entrapment Efficiency (%)
Percentage of Entrapment Efficiency (%)
Percentage of Loading Efficiency (%)
16
32
64
100
200
Concentration of Rapamycin (μg/mL)

Figure S5

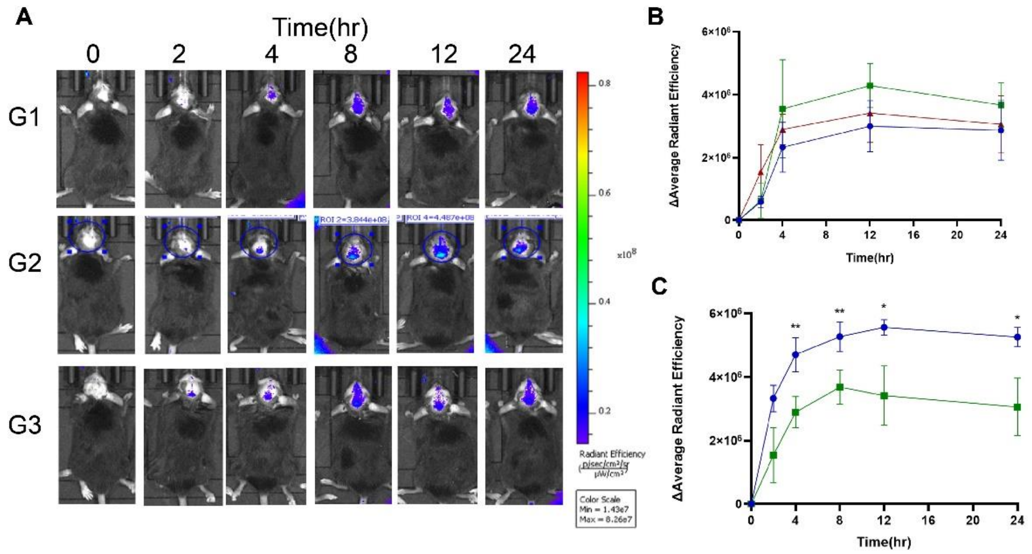

A
Time(hr)
0
2
4
8
12
24
G1
G2
G3
Radiant Efficiency
Color Scale
Min = 1.43e7
Max = 8.26e7
B
ΔAverage Radiant Efficiency
Time(hr)
C
ΔAverage Radiant Efficiency
Time(hr)

Figure S6

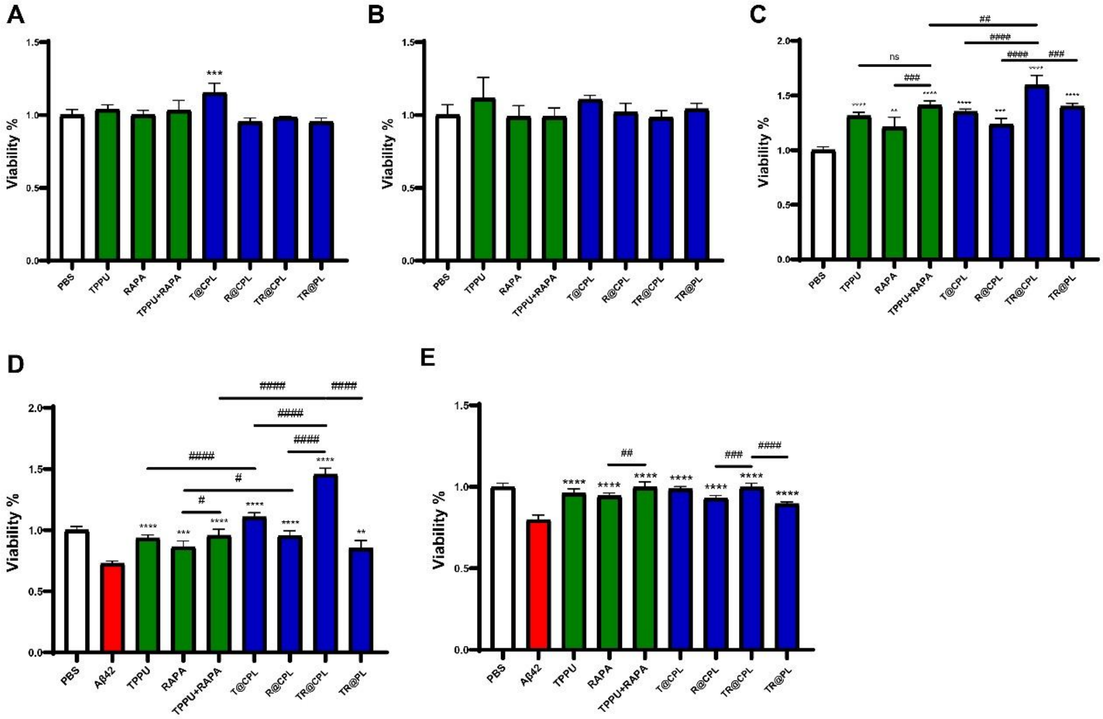
A
B
C
D
E
Viability %
PBS
Aβ42
TPPU
RAPA
TPPU+RAPA
T@CPL
R@CPL
TR@CPL
TR@PL
ns
#
##
###
####
**
***
****

Figure S7

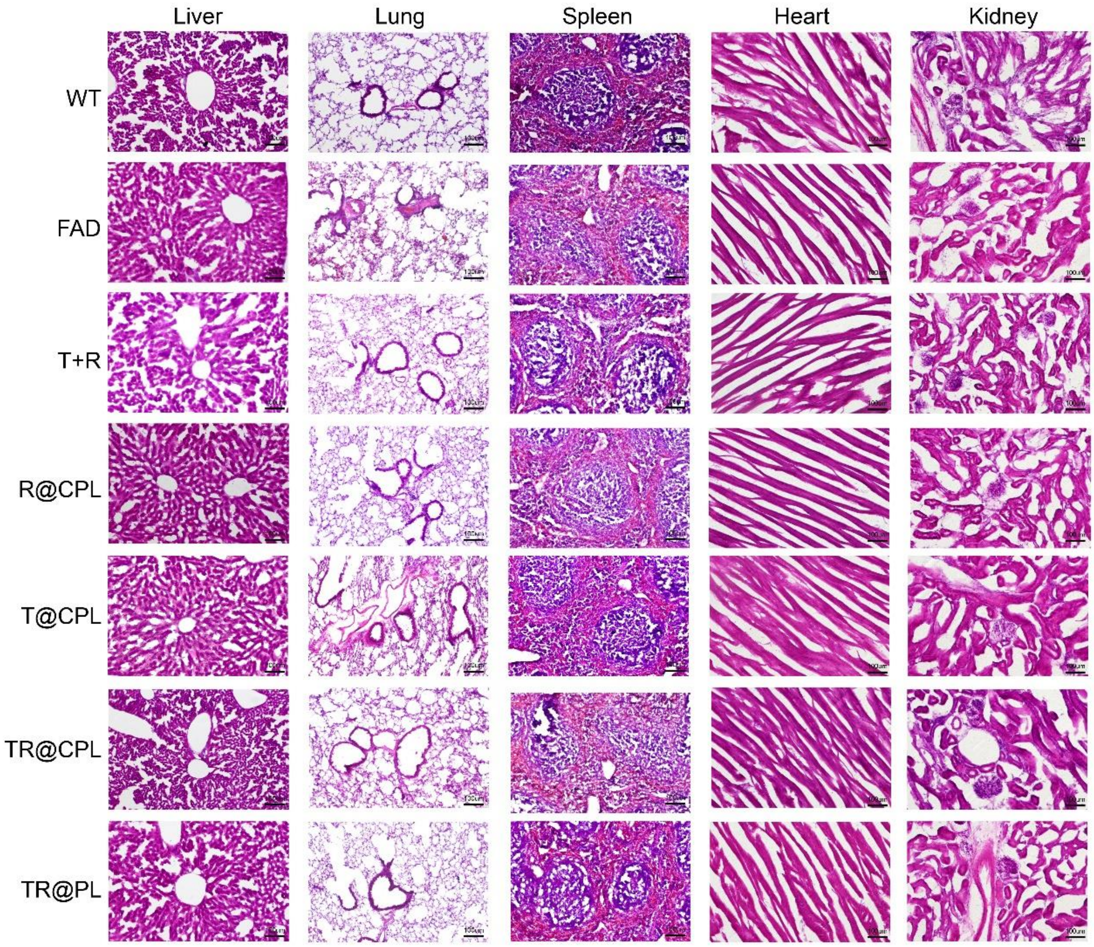
Liver
Lung
Spleen
Heart
Kidney
WT
FAD
T+R
R@CPL
T@CPL
TR@CPL
TR@PL
